# Treatment of Dentine Hypersensitivity by Diode Laser: A Clinical Study

**DOI:** 10.1155/2012/858950

**Published:** 2012-06-25

**Authors:** Romeo Umberto, Russo Claudia, Palaia Gaspare, Tenore Gianluca, Del Vecchio Alessandro

**Affiliations:** Department of Oral and Maxillofacial Sciences, Sapienza University of Rome, Via Caserta 6, 00161 Rome, Italy

## Abstract

*Introduction*. Dentine hypersensitivity (DH) is characterized by pain after stimuli that usually provoke no symptoms. This study compared the effectiveness of GaAlAs diode laser alone and with topical sodium fluoride gel (NaF). *Materials and Methods*. The study was conducted on 10 patients (8 F/2 M, age 25–60) and 115 teeth with DH assessed by air and tactile stimuli measured by Numeric Rating Scale (NRS). Teeth were randomly divided into G1 (34 teeth) treated by 1.25% NaF; G2 (33 teeth) lased at 0.5 W PW (T on 100 m and T off 100 ms), fluence 62.2 J/cm^2^ in defocused mode with a 320 **μ** fiber. Each tooth received three 1′ applications; G3 (48 teeth) received NaF gel plus laser at same G2 parameters. NRS was checked at each control. *Results*. Significant pain reduction was showed. The NRS reduction percentages were calculated, and there was a concrete decrease of DH above all in G3 than G2 and G1. *Conclusion*. Diode laser is a useful device for DH treatment if used alone and mainly if used with NaF gel.

## 1. Introduction

Dentine hypersensitivity (DH) is an abnormal response of the exposed vital dentine to thermal, chemical, or tactile stimuli. The prevalence of DH has been reported ranging from 4 to 57% in many studies in the literature, depending on the population samples studied [1, 2]. In patients affected by periodontitis, DH prevalence was even higher ranging between 60 to 98% [3]. However, DH prevalence is likely to increase in next years since more adults keep their teeth into later life. This condition may affect patients at any age, and both genders are equally affected [4, 5].

Pain of dentinal origin is sharp, localized, and of short duration. Although different theories have been proposed for the mechanism involved in DH etiology, recent studies gave support to Brannstrom's hydrodynamic theory [6], according to this a stimulus applied to open tubules dentin increases the flow of dentinal tubular fluid, with mechanical deformation of the nerves located into the inner ends of the tubules or in the outer layers of the pulp [7]. Type A delta fibers are supposed to be responsible for dentinal sensitivity being probably activated by the hydrodynamic process [8].

The most common factors involved in DH are abrasion, caused by inadequate intensity tooth brushing; abfraction, caused by teeth flexion due to abnormal occlusal forces; parafunctions or occlusal disequilibrium; erosion, secondary to the presence of acids in the oral cavity, as in bulimia nervosa or gastroesophageal reflux; anatomic predisposition due to structural deficiency of the enamel-cement junction; cavity preparations in vital teeth that expose dentine or badly controlled dentinal acid conditioning [8–10].

Orchardson and Gillam showed that DH affects above all the vestibule-cervical area of teeth [4]. Cervical DH has probably a multifactorial etiology, and more than a cause is related to this painful manifestation. Therefore, several treatments must be associated to reduce DH to satisfactory levels. According to Garone-Filho [10] the abfraction, caused by occlusal overload, is the most common etiological factor related to DH. So an occlusal adjustment should be always associated to the treatment of DH. 

Furthermore, according to Pashley [11], there are two types of dentinal permeability: intratubular, into the dentinal tubules, and intertubular, between the tubules in dentinal matrix. The sensitive dentine is permeable through its thickness; any treatment that reduces dentinal permeability must reduce dentinal sensitivity. The greatest dentine diffusion capacity allows the best interaction with the desensitizing agent. In fact, occlusion of the exposed dentinal tubules may decrease dentinal sensitivity level [9–14]. However, the DH sometimes persists despite of the effective sealing of the tubules, so indicating that further mechanisms are involved in nerves activation instead of or in addition to the hydrodynamic mechanism. Many authors suggested the hypothesis concerning the release of neuropeptides from the activated nervous terminations and, subsequently, the induction of a neurogenic inflammation. This hypothesis should signify that the symptoms of DH could become self-sustainable up to a certain point [8, 14].

Another great problem related to DH is its evaluation, since pain is a highly subjective sensation. Nevertheless, it is possible to classify the DH according to Matsumoto's criteria. In this classification, three degrees of DH are recognized: grade 1 mild discomfort/pain, grade 2 moderate pain, and grade 3 characterized by intense and unbearable pain [15]. 

Through literature examination emerges that there is no therapy that can always reduce pain at satisfactory levels, even with the combination of different protocols. According to Landry and Voyer [16], there is not an ideal desensitizing agent but any kind of treatment for DH should be effective from the first application and must satisfy these parameters established by Grossman since 1934 [17]: (1) not irritating pulp, nor causing pain, (2) easy application, (3) long-lasting effect, (4) not discoloring or staining teeth, (5) not irritating soft tissues or periodontal ligament, (6) low cost.

Every treatment, that reduces dentinal permeability, diminishes dentinal sensitivity. The occlusion of dentinal tubules leads to the reduction of dentinal permeability so decreasing the degree of DH [11]. According to the hydrodynamic theory, the effectiveness of dentine desensitization agents is directly related to their capacity of promoting the sealing of the dentinal canaliculi [12].

Conventional therapies for DH are based on the local application of desensitizing agents, either professionally or at home. The most frequently used agents can be classified as protein precipitants [18], tubule-occluding agents [19, 20], tubule sealants [21]. The sodium fluoride gel (NaF), which belongs to the tubule-occluding agents family, is the most commonly used agent [4, 22–25]. Its mechanism relies on the mechanical occlusion that is accomplished by precipitation of insoluble calcium fluoride crystals within the tubules without adhesion. For this reason, it cannot resist to the stresses of the oral environment and its action decreases with time [4, 23].

In the last fifteen years, the introduction of lasers gave further possibilities to DH therapy [22, 26–29]. Laser photobiomodulating action in dental pulp was reported by many authors as in Villa et al. [30], with histological studies of dental pulp of mice, after laser irradiation in teeth with exposed dentine. In this study, the authors registered a large quantity of tertiary dentine production in lased teeth, that caused the physiological obliteration of tubules, while the nonirradiated control showed intense inflammatory process that, in some cases, evolved into necrosis. Focusing on the role of laser in DH therapy, it is possible to show that its action is twofold. By one side, the low-level power lasers [14, 31], also called “soft lasers,” act directly on nerve transmission, with a depolarization process that prevents the diffusion of pain to SNC; however, their effectiveness seems poorer in higher degrees of DH. By the other side, high-power lasers such as: diode 980 nm and 808 nm, KTP 532 nm, Nd: YAG 1064 nm, CO_2_ 10600 nm, Er, Cr: YSGG 2780 nm, and Er: YAG 2940 nm act on DH provoking a melting effect with crystallization of dentine inorganic component and the coagulation of fluids contained into the dentinal tubules. Among these “high power” devices, diode lasers are the most studied and the ones that gave the best results in several clinical protocols even in high-grade DH cases. 

The aim of this study is to assess the efficacy of a diode GaAlAs laser alone and in combination with topical sodium fluoride gel (NaF) in the treatment of DH in order to evaluate the possibilities of this device in the management of this painful condition.

## 2. Materials and Methods

**Figure 1:**
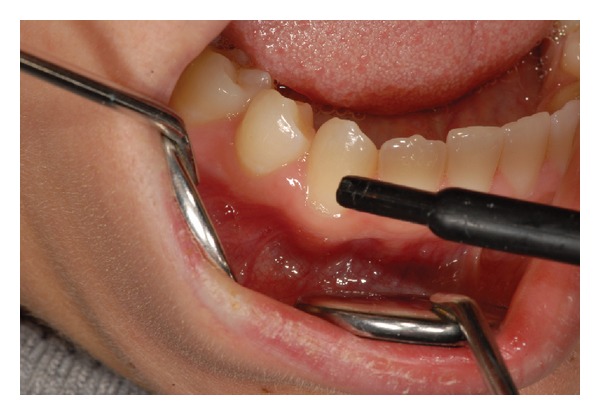
Air stimulus application.

**Figure 2:**
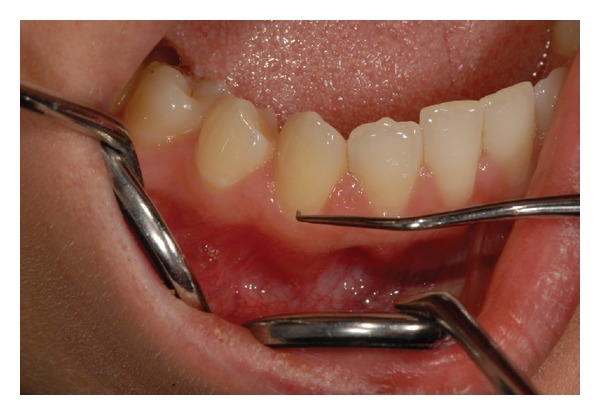
Tactile stimulus application.

The study was conducted on 10 patients (8 females and 2 males; aged from 25 to 60 years) and in a total of 115 teeth with DH assessed by mean of both air (Figure 1) and tactile (Figure 2) stimuli measured by the Numeric Rating Scale (NRS). 

The inclusion criteria for patient enrollment were based on: the absence of local (e.g., cavities, fractures) and/or systemic pathologies, on the absence of contraindications to the proposed therapies (e.g., allergies to desensitizing agents) and on the presence of teeth with DH evaluated by pain response to both air and tactile stimuli that were registered by NRS scale (from 0 to 10, where 0 meant the absence of pain and 10 represented an unbearable pain and discomfort felt by the patients in their life); at last no desensitizing therapy had to be previously performed, nor analgesic drugs had to be recently assumed. 

Before any treatment, all the patients received a hygiene professional program with oral hygiene instructions and the teeth vitality of all sites was assessed.

**Figure 3:**
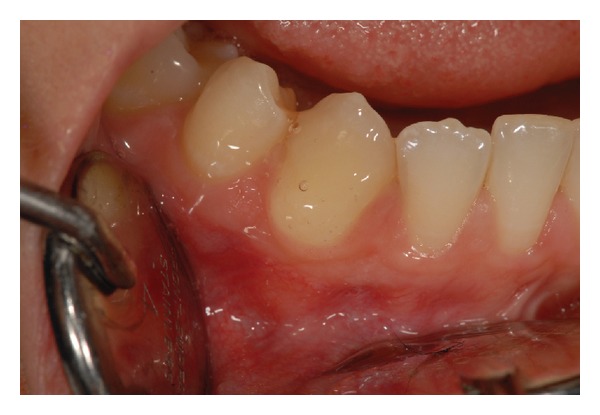
NaF gel application (Group 1).

**Figure 4:**
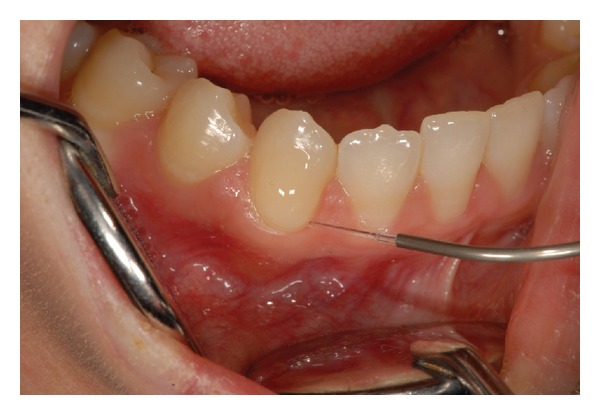
GaAlAs laser application (Group 2).

**Figure 5:**
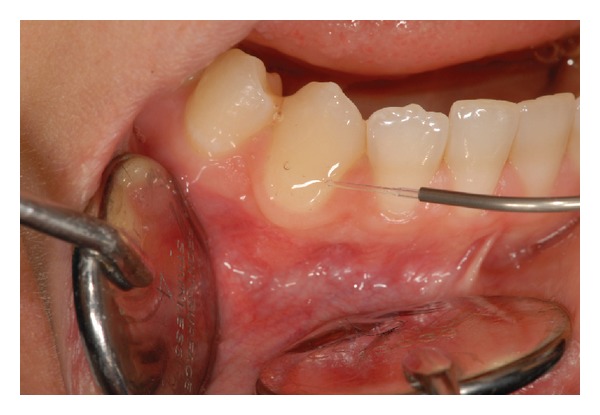
GaAlAs laser application + NaF gel application.

For each patient, the sensitive sites were randomly divided into three groups:

- Group 1 (G1) (34 teeth) treated with 1.25% NaF applied for 60 seconds on tooth surface (Figure 3);
- Group 2 (G2) (33 teeth) lased by a GaAlAs laser (DoctorSmile, Lambda S.p.A., Brindole (Vi), Italy, 980 nm) with these parameters: 0.5 W in PW (T on 100 ms and T off 100 ms) and fluence of 62.2 J/cm^2^ in no contact mode and using a fiber of 320-micron diameter. Each site received 3 applications of 1 minute each (Figure 4) once a week for three weeks;
- Group 3 (G3) (48 teeth) treated using both NaF gel and diode laser at the same parameters of G2. The NaF gel was left on tooth surface for 60 seconds before the irradiation; in this way, the laser system could favor the permanence of desensitizer for a longer time than when it was used alone (Figure 5).

Patients' response to cold air blast was assessed by a short blast of 1-second duration at a distance of 0.5 cm for each tooth. Both air and tactile stimuli evaluations were performed before and after every treatment session, for a total of 3 treatment sessions at a distance of about one week each other.

The obtained results have been statistically analyzed through the Graphpad Prism 5.0 software.

## 3. Results

All the groups registered significant improvements of discomfort. A reduction of DH occurred during the treatment sessions, and the positive values were maintained after 1 month (Tables 1, 2, and 3).

Comparing the three regimens, a higher decrease of DH was registered in G3, followed by G2 and G1, respectively, whose results seem to be almost superimposable. The NRS reduction percentage was valued for each group between the first pretreatment and the third posttreatment session (Immediate−/−). The values were divided depending on the kind of stimulation. For the air stimulus, the reduction percentage was, respectively, 10.19% (I) for G1; 22.35% (I) for G2; 25.04% (I) for G3. Furthermore, the tactile stimulus took down: 4.13% (I) for G1; 6.77% (I) for G2; 9.96% (I) for G3.

**Figure 6:**
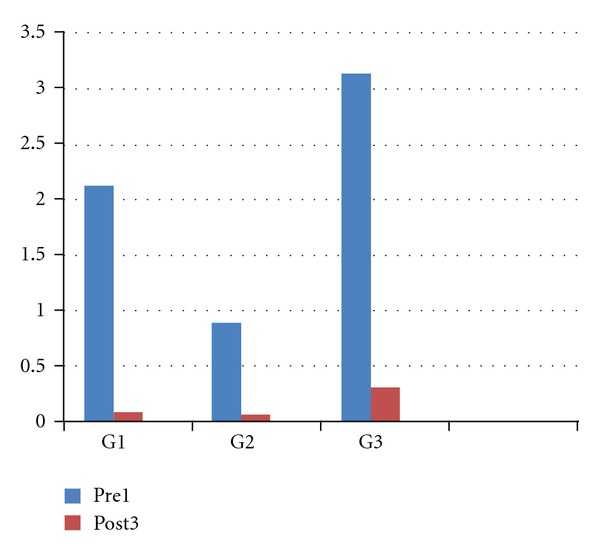
Illustrative representation of the improvements to the tactile stimuli from the first treatment to the third one.

Regarding to the statistical analyses, the data relating to the probe test (Figure 6) were subjected to the Kruskal-Wallis's test which demonstrated the reliability of the study (*P* < 0.0001). The comparative Dunn's test showed a statistically significant difference in G3 (*P* < 0.001), and in G1 (*P* < 0.01). In G2 the obtained improvement were lesser statistically significant (*P* < 0.1).

**Figure 7:**
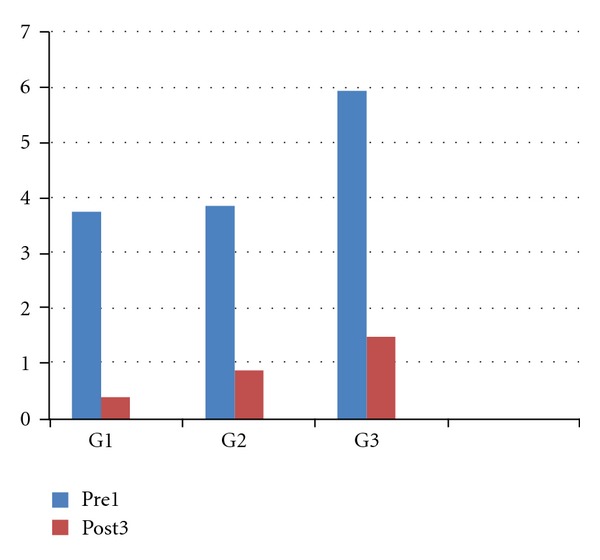
Illustrative representation of the improvement to the air stimuli from the first treatment to the third one.

The results obtained with the cold blast (Figure 7) air were always analyzed through the Kruskal-Wallis's test which demonstrated the reliability of the study (*P* < 0.0001) and the comparative Dunn's test showed a statistically significant difference in each treated group (*P* < 0.001).

The improvement obtained in the samples of the probe test was not statistically significative (*P* > 0.05) instead of the samples of the cold blast air in which the improvements from the first treatment to the last one were superimposable. 

## 4. Discussion

Through literature examination, it is clarified that the ideal treatment for DH does not exist, even in case of combination of different protocols.

Conventional therapies for the treatment of DH comprehend the topical use of desensitizing agents, either professionally or at home such as protein precipitants [18], tubule-occluding agents [19, 20], tubule sealants [21], and, recently, lasers [22, 26–29].

Several studies [32–34] describe a synergistic action of lasers in association with desensitizing agents. In fact, the laser system can favor the permanence of the desensitizer for longer time than when they are used alone. For this reason, if laser device is used in addition to a conventional desensitizing agent, the latter remains above the tooth surface for 60 seconds before the irradiation.

Focusing on the effectiveness of the sole diode laser, this was investigated by several authors. Matsumoto et al. [35] showed an 85% improvement in teeth treated with laser; Aun et al. [36] reported success in laser-irradiated teeth in 98% of their cases; Yamaguchi et al. [37] noticed an effective improvement index of 60% in the group treated with laser compared to the 22.2% of the control nonlased group; Kumazaki et al. [38] showed an improvement of 69.2% in the group treated with laser compared to 20% in the placebo group. Gerschman et al. [39], in a double-blind study, found significant values in the laser-treated group. In fact, sensitivity to thermal stimuli was reduced by 67%, whereas the placebo group had a reduction of 17%, sensitivity to tactile stimuli was reduced by 65%, while the placebo group showed a reduction of 21%. Another study carried out by Brugnera et al. [40] showed the immediate analgesic effect using a diode laser. 

In this study, significant improvements in pain and discomfort were always registered after the session treatments (I) even if in no case the percentages of pain reduction arrived to the high values registered in the literature.

As a first laser showed the best immediate results alone and in combination with gel, since the percentages of pain reduction in G2 and G3 were more than twice than G1 values. In our sample, the best results were obtained by the association of laser and NaF gel therapy (G3). This group registered the highest I reduction, in particular for air blast stimulation. It is probable that the better performance of combined treatment was due to the higher NaF gel adhesion to the dentinal tubules when combined with laser energy. In the laser group, G2, the immediate pain reduction was very high especially at air stimulation (22.35% I); in the same group, the improvement at tactile stimulation was poorer after the treatment (6.77% I). The lower reduction values were registered in the sole gel group, G1, by both stimulations, in the immediate period.

Even in consideration of the short sample analyzed, it is possible to speculate that the laser-induced superficial melting permits to keep longer the tubules occlusion by NaF gel emphasizing the reduction of DH-related pain.

## 5. Conclusion

According to these results, the GaAlAs laser showed a very high capability to improve immediately the DH-related pain, both alone and even better in combination with NaF gel. On the other hand, the sole gel results, even if positive, cannot equalize the performances of laser in the immediate. These results have to be confirmed by greater samples of patients and by longer follow-up periods (e.g., 3 and 6 months) to confirm or not the long-lasting action of the combined laser and gel therapy.

## Figures and Tables

**Table 1:** Chart of NRS pretreatment, posttreatment, and at 1-month control values of the G1 (only NaF gel).

Preair	Postair	Air control	Preprobe	Postprobe	Probe control
evaluation	evaluation	1 month	evaluation	evaluation	1 month
4	7	0	3	1	0
5.5	0	0	6.5	0	0
5.5	0	0	3	0	0
10	2	0	6	0	0
10	0	0	10	0	0
5.5	0	0	5	1	0
3	0	0	5	1	0
3	0	0	3	0	0
4	0	0	0	0	0
3	0	1	0	0	0
1	0	2	0	0	0
1	0	0	0	0	0
1	0	2	1	0	0
2	0	0	0	0	0
2	0	0	0	0	0
2	0	0	0	0	0
1	0	0	0	0	0
0	0	2	6	0	0
0	0	0	6	0	0
7	1	0	7	0	0
4	1	1	0	0	0
1	0	0	0	0	0
1	0	0	0	0	0
1	0	0	0	0	0
1	0	0	0	0	0
0	0	0	2.5	0	0
0	0	0	4	0	0
2	0	0	0	0	0
3	0	0	0	0	0
10	0	0	0	0	0
10	2	0	4.5	0	0
5	0	0	0	0	0
5	0	0	0	0	0
5	0	0	0	0	0

**Table 2:** Chart of NRS pretreatment, posttreatment, and at 1-month control values of the G2 (only Diode laser).

Preair	Postair	Air control	Preprobe	Postprobe	Probe control
evaluation	evaluation	1 month	evaluation	evaluation	1 month
6	0	1	5	0	0
2	0	0	0	0	0
3	0	0	2.5	0	0
4	0	0	1.5	1	0
8	4	0	3	0	0
8	4	0	3	0	0
3	0	0	0	0	0
1	0	0	0	0	0
1	0	0	3.5	0	0
4	3	1	0	0	0
5	1	0	0	0	0
4	0	1	0	0	0
4	1	3	0	0	0
2	0	0	0	0	0
5	2	1	2	0	0
2	2	2	0	1	2
1	0	0	0	0	0
2	0	0	0	0	0
5	0	0	0	0	0
7	0	0	3	0	0
1	0	0	0	0	0
1	0	0	0	0	0
1	0	0	0	0	0
3	0	0	3	0	0
3	0	2	3	0	0
2	0	0	0	0	0
6.5	0	0	0	0	0
8	3.5	0	0	0	0
5	3	0	0	0	0
5	0	0	0	0	0
5	2	0	0	0	0
5	0	0	0	0	0
5	3	0	0	0	0

**Table 3:** Chart of NRS pretreatment, posttreatment, and at 1-month control values of the G3 (NaF gel + Diode laser).

Preair	Postair	Air control	Preprobe	Postprobe	Probe control
evaluation	evaluation	1 month	evaluation	evaluation	1 month
7	0	0	4	0	0
7	0	4	1	0	0
8	8	8	9	7	4
2	0	0	7	0	0
2	0	0	0	0	0
8	7	5.5	9	0	0
5	0	3	5	0	0
9	4	6	9	0	0
7	0	7	7	3	0
4	5	3	8	0	2
4	2	3	0	0	0
7	0	0	9	0	0
7	0	0	7	0	0
10	1	3	10	2	0
6	0	0	3	0	0
8	0	0	8	0	0
10	10	0	10	1	0
5	1	0	3.5	0	0
3	0	0	1.5	0	0
3	0	0	3	0	0
9	0	0	9	0	0
10	5	0	1.5	0	0
10	7	0	3	0	0
5	1	2	0	0	0
6	2	0	0	0	0
10	0.5	0.5	2	0	0
10	1	0	2	0	0
10	1	0	2	0	0
2	0	0	0	0	0
3	2	0	2	0	0
3	2	0	4	1	0
4	0	0	4	1	0
2	0	0	0.5	0	0
5	0	0	0	0	0
9	0	2	0	0	0
8	1	0	8	0	0
1	0	0	0	0	0
3	0	0	0	0	0
3	0	0	0	0	0
1	0	0	1	0	0
10	0	0	0	0	0
10	0	0	0	0	0
0	0	0	2.5	0	0
5	0	0	0	0	0
5	4	0	0	0	0
10	4	0	0	0	0
8	3	0	0	0	0
5	0	0	0	0	0
